# Supplementary material for: Self-Reported Medication Use among Pregnant and Postpartum Women during the Third Wave of the COVID-19 Pandemic: A European Multinational Cross-Sectional Study
Source: Int J Environ Res Public Health. 2022 Apr 27;19(9):5335. doi: 10.3390/ijerph19095335 (PMC9100043; doi:10.3390/ijerph19095335)
Supplement: Supplementary file 1 [file ijerph-19-05335-s001.zip › ijerph-1691386-supplementary.pdf]

Supplementary Materials

Supplementary Materials S1. English version of the survey relevant to this manuscript**.**

Supplementary Materials S2. Recruitment tools utilized and internet penetration rates.

Supplementary Materials S3. Overview of the infection rates and imposed regulations in each country during the study period of 10 June until 22 August 2021.

Supplemental Table S1. Prevalence of the use of supplements/vitamins among pregnant and post-partum women, by country.

Supplemental Table S2. The ten most reported ATC classes and individual medications within each class among pregnant women according to country.

Supplemental Table S3. The ten most reported ATC classes and individual medications within each class among post-partum women according to country.

Supplemental Table S4. Overview of the ten most reported ATC classes of chronic medications changed on women's own initiative due to the pandemic.

Supplemental Table S5. Categorization of reasons for changing the use of a chronic medication due to the COVID-19 pandemic into thematic key concepts.

Supplemental Table S6. Comparison between the general birthing population and the study sample of pregnant and postpartum women for each country.

Supplementary Materials S1. English version of the questionnaire relevant to this manuscript.

1. Did you participate in the previous COVID-survey distributed in June-July 2020?
a. Yes
b. No
c. I don’t know

Pregnancy-specific

2. How many weeks pregnant are you right now? (dropdown menu: 0-42)

3. Was the pregnancy planned?
a. Yes
b. No
c. No, but it was not unexpected

4. Have you been pregnant before?
a. Yes
b. No

5. If yes to Q4: How many children do you already have (not including the current pregnancy)?

Postpartum-specific

6. Please indicate the age of your baby.
a. 6 weeks
b. Between 6 weeks and 12 weeks

7. Was your child admitted to the neonatal intensive care unit (NCU) after the delivery?
a. Yes
b. No

8. Are you currently breastfeeding and/or giving expressed milk?

a. Yes
b. No

If currently breastfeeding:

9. Please indicate which applies:
a. My baby only receives breastmilk (via breastfeeding or expressed breastmilk)
b. My baby receives both breastmilk and solid food or formula feeding

10. Did you ever breastfeed / give expressed milk before the coronavirus outbreak?
a. Yes
b. No

11. If yes to Q6, did the coronavirus pandemic have an impact on your breastfeeding compared to the previous time before the pandemic?
a. Yes
b. No

12. If yes to Q7: Please explain how the coronavirus has affected your breastfeeding (open text field, optional question).

13. To which extent have you considered not to breastfeed/give expressed milk due to the coronavirus pandemic?
a. Not considered at all
b. Barely considered
c. Slightly considered
d. Strongly considered

14. To which extent have you considered breastfeeding/giving expressed breastmilk more or for a longer period of time?
a. Not considered at all
b. Barely considered
c. Slightly considered
d. Strongly considered

If currently not breastfeeding:

15. Did you breastfeed your baby and/or give expressed milk during the last three months?
a. Yes
b. No

16. If yes to Q5: Did you stop breastfeeding and/or giving expressed milk due to the coronavirus pandemic?
a. Yes
b. No

Coronavirus infection

In this section, you will be asked whether you and members of your household / close family have been infected with the new coronavirus and how it was diagnosed.

17. Did you have a positive test for coronavirus infection?
a. Yes
b. No

18. If no to Q6: Do you believe you were infected with the coronavirus although not confirmed by a test?
a. Yes
b. No

19. Has any member(s) of your household or close family tested positive for the coronavirus since the start of the pandemic?
a. Yes
b. No

Covid-19 and disease severity

In this section, you will be asked how COVID-19 affected you and the members of your household / family personally.

20. If a) to Q6 or Q8: In what way do/did your symptoms due to COVID-19 influence your normal functioning?
a. No influence at all
b. Limited influence
c. Rather large influence

d. Very large influence

Covid-19 and pregnancy

In this section you will be asked about your perception about COVID-19 disease severity during pregnancy.

21. Do you believe coronavirus infection can be more severe during pregnancy than among non-pregnant women of the same age?
a. Yes
b. No

22. If yes to Q13: can you specify why you believe a coronavirus infection can be more severe during pregnancy (multiple answers possible):
a. I believe pregnant women are at increased risk of more severe disease with coronavirus infection (for example intensive care unit admission) due to the underlying pregnancy state
b. I believe coronavirus infection during pregnancy can cause malformations to the unborn child
c. I believe coronavirus infection during pregnancy can affect the growth and development of the unborn child
d. I believe coronavirus infection during pregnancy increases the risk of pregnancy complications (for example preterm birth)
e. I believe coronavirus infection could be transmitted to my baby during delivery
f. Other reason: ….

Coronavirus and postpartum

23. To what extent do you believe that breastfeeding can be risky for the nursing infant if a breastfeeding mother is infected with coronavirus?
a. Extremely risky
b. Very risky
c. Risky
d. Not very risky
e. Not risky at all

24. If a, b or c to Q18: Can you specify why you believe that breastfeeding can be risky for the nursing infant if a breastfeeding mother is infected with the coronavirus (multiple answers possible):

a. I believe the virus can be transmitted to the baby through milk
b. I believe the baby could be infected while breastfeeding (through close contact)
c. I believe the disease could stop the mother from breastfeeding (feeling too sick)
d. I believe the disease could negatively affect the milk production
e. Other

Coronavirus and your wellbeing

25. In this section, you will be asked about the impact of COVID-19 pandemic on your mental health and wellbeing.

	Not at all anxious	Slightly anxious	Anxious	Very anxious	Extremely anxious	Not applicable
a. Disrupted antenatal appointments						
b. Lack of information or inconsistent information from healthcare professionals						
c. Isolation						
d. Lack of antenatal classes						
e. The thought of not having my partner/ birth partner with me during birth						
f. The thought of my partner having to leave the hospital soon after birth						
g. The thought of being separated from my baby after delivery						
h. Not having support from family and friends						

We would like to know how you are feeling. Please indicate the answer, which comes closest to how you have felt in the past 7 days, not just how you feel today.

In this section of the questionnaire - the Edinburgh Postnatal Depression Scale (EPDS) was presented (Cox J, Holden J, Sagovsky R. Detection of postnatal depression. Development of the 10-item Edinburgh postnatal depression scale. The British Journal of Psychiatry. 1987 June 1, 1987;150(6):782-6).

Please indicate the answer which comes closest to how you have felt in the past 14 days.
Over the last 14 days, how often have you been bothered by the following problems?

In this section of the questionnaire - Generalized Anxiety Disorder 7-item scale (GAD-7) was presented (Spitzer RL, Kroenke K, Williams JBW, Löwe B. A Brief Measure for Assessing Generalized Anxiety Disorder: The GAD-7. Archives of Internal Medicine. 2006;166(10):1092-7).

43. Please indicate for which chronic illnesses you have used a medication in the past 3 months? (i.e., chronic illnesses are conditions that already existed before your pregnancy/postpartum) (multiple answers possible)?

a. Asthma
b. Allergy
c. Cardiovascular disease (e.g., high blood pressure, high cholesterol, heart disease, etc.)
d. Depression
e. Diabetes
f. Epilepsy
g. Hypothyroidism (underactive thyroid)
h. Rheumatic diseases (including rheumatoid arthritis, psoriatic arthritis, etc.)
i. Inflammatory bowel disease (including Crohn’s disease, Ulcerative colitis, etc.)
j. Anxiety
k. Other: ….
l. None of the above

44. If answer yes to Q52: Please indicate for which symptoms/conditions you have used a medication in the past 3 months (multiple answers possible)?

a. Pain
b. Migraine
c. Fever
d. Infection
e. Nausea or vomiting

f. Gastro esophageal reflux disease / heartburn
g. Constipation
h. Other: …..
i. None of the above

Personal background

In this final section, you will be asked a few questions about yourself as a participant of this study.

45. Year of birth? (Dropdown menu)

46. Which language do you (primarily) speak at home? (Dutch, French, German, English, Norwegian, Arabic, other…)

47. Where do you live? (country-specific adaption is needed for this question)

48. Do you currently have a partner?
a. Yes
b. No

49. Did you smoke before you got pregnant?
a. Yes
b. No

50. Have you smoked since you found out you were pregnant?
a. Yes
b. No

51. Please indicate how often you have drunk alcohol since you found out you are pregnant.
a. at least 2 times per week

b. 2 to 4 times per month
c. Monthly or less
d. Once
e. Never

52. Did you take folic acid during your pregnancy?
a. Yes
b. Yes, and I had already started using it before my pregnancy
c. No

53. What is your highest level of education?
a. Primary education
b. Professional secondary education
c. Technical secondary education
d. Artistic secondary education
e. General secondary education
f. Professional bachelor
g. Academic bachelor
h. Master
i. PhD
j. Other: ……….

54. What is your current professional status? (if you are currently not working because of pregnancy complications or maternity leave, please fill in the professional status you had before)
a. Employee
   Are/were you working in healthcare?
   Does your employment put you at increased risk of catching COVID-19 ?
b. Self-employed
   Are/were you working in healthcare?

Does your employment put you at increased risk of catching COVID-19 ?

c. Civil service employee

Are/were you working in healthcare?

Does your employment put you at increased risk of catching COVID-19 ?

d. Student

Are you following an education in healthcare?

e. Homemaker

f. Jobseeker/unemployed

g. Incapacitated/disabled

h. Other: ....

Are/were you working in healthcare?

Does your employment put you at increased risk of catching COVID-19 ?

55. Finally, the coronavirus pandemic affected many people in many different ways, both negatively and/or positively. Please share any of your personal experiences or anything that has changed in your life due to the pandemic (optional question).

Supplementary Materials S2. Recruitment tools utilized and internet penetration rates.

Country	*Websites used for recruitment PLEASE CHECK*	*Internet penetration rates (%)*
Norway	www.altformamma.no (i.e. pregnancy website); several Facebook sites (e.g. Facebook: Gravid i coronatider); pregnancy forums (e.g. Facebook: Termin september 2020); mobile application (Helseappen).	99.0 [1]
Belgium	www.gezondzwangerworden.be; several Facebook pages and websites of perinatal organizations, midwives, lactation consultants, community pharmacies,…	96.0[2]
Switzerland	www.chuv.ch; www.swissmom.ch; several Facebook pages;	95.0[3]
The Netherlands	www.consumentenbond.nl; www.24baby.nl; several Facebook sites (e.g. ik ben zwanger, kindje klein); website for midwives (www.deverloskundige.nl); Lareb website (www.lareb.nl and www.lareb.nl/tis-knowledge).	94.2[4]
UK	www.medicinesinpregnancy.org (i.e. the UKTIS patient facing website); Twitter (UKTIS account); Facebook (local maternity/mother and baby groups).	99.3[5]

Sources of internet penetration rates: [1] Statistics Norway (https://www.ssb.no/en); [2] Statistics Flanders, Belgium (www.statistiekvlaanderen.be); [3]Statistics Switzerland (www.bfs.admin.ch); [4]Statistics Netherlands (www.statistica.com); [5] Statistics UK (https://www.ons.gov.uk)

Supplementary Materials S3. Overview of the infection rates and imposed regulations in each country during the study period of 10 June until 22 August 2021.

**Country**	**Data Collection Time Period (2021)**	**First reported COVID-19 Case/Death (2020)**	**Infection Rates as Inhabitants Infected/Day (July 1st 2021)**	**Percentage of the Population (>18 years) with One Vaccination Dose (1 Dose/2 Doses) (July 1st 2021)**
Norway	01.07 to 24.07	26 Feb/12 Mar	192	51% / 29%
Belgium	10.06 to 18.07	04 Feb/11 Mar	670	64% / 44%
Switzerland	14.06 to 22.08	27 Feb/6 Mar	172	51% / 37%
The Netherlands	11.06 to 18.07	27 Feb/6 Mar	1044	72% / 41%
UK	14.06 to 30.07	31 Jan/5 Mar	26527	85% / 63%

Supplemental Table S1. Prevalence of the use of supplements/vitamins among pregnant and post-partum women, by country.

	Pregnant Women						Post-Partum Women					
	**Overall** (N=3411) *N (%)*	**Norway** (N=2376) *N (%)*	**Belgium** (N=360) *N (%)*	**Switzerland** (N=210) *N (%)*	**UK** (N=290) *N (%)*	**Netherlands** (N=175) *N (%)*	**Overall** (N=1799) *N (%)*	**Norway** (N=1113) *N (%)*	**Belgium** (N=235) *N (%)*	**Switzerland** (N=176) *N (%)*	**UK** (N=120) *N (%)*	**Netherlands** (N=155) *N (%)*
**Multivitamins**	504 (14.8)	339 (14.3)	62 (17.2)	39 (18.6)	43 (14.8)	21 (12)	252 (14.0)	201 (18.1)	30 (12.8)	12 (6.8)	4 (3.3)	5 (3.2)
**Folic acid**	463 (13.6)	278 (11.7)	68 (18.9)	32 (15.2)	55 (19.0)	30 (17.1)	115 (6.4)	86 (7.7)	6 (2.6)	8 (4.6)	7 (5.8)	8 (5.2)
**Iron-containing preparations**	288 (8.4)	228 (9.6)	19 (5.3)	19 (9.1)	16 (5.5)	6 (3.4)	171 (9.5)	125 (11.2)	14 (6.0)	7 (4.0)	16 (13.3)	9 (5.8)
**Omega-3 containing products**	157 (4.6)	138 (5.8)	7 (1.9)	7 (3.3)	2 (0.7)	3 (1.7)	83 (4.6)	79 (7.1)	1 (0.4)	2 (1.1)	1 (0.8)	0 (0.0)
**Other products**	294 (8.6)	197 (8.3)	35 (9.7)	142 (67.6)	13 (4.5)	16 (9.1)	122 (6.8)	91 (8.2)	9 (3.8)	11 (6.3)	6 (5.0)	5 (3.2)

Supplemental Table S2. The ten most reported ATC classes and individual medications within each class among pregnant women according to country.

**Medication Class**	**Any Use (N = 3411)**	**Norway (N=2376)**	**Belgium (N=360)**	**Switzerland (N=210)**	**United Kingdom (N=290)**	**Netherlands (N=175)**
Analgesics (N02)	470 (13.8)	152 (6.4)	129 (35.8)	49 (23.3)	97 (33.4)	43 (24.6)
Paracetamol (N02BE01)	355 (10.4)	123 (5.2)	98 (27.2)	38 (18.1)	68 (23.4)	28 (16.0)
Antihistamines for systemic use (R06)	248 (7.3)	101 (4.3)	73 (20.3)	30 (14.3)	23 (7.9)	21 (12.0)
Cetirizine (R06AE07)	62 (1.8)	28 (1.2)	18 (5.0)	9 (4.3)	2 (0.7)	5 (2.9)
Drugs for acid related disorder (A02)	243 (7.1)	55 (2.3)	72 (20.0)	20 (9.5)	63 (21.7)	33 (18.9)
Omeprazole (A02BC01)	50 (1.5)	10 (0.4)	12 (3.3)	4 (1.9)	16 (5.5)	8 (4.6)
Antithrombotic agents (B01)	116 (3.4)	46 (1.9)	27 (7.5)	5 (2.4)	29 (10.0)	9 (5.1)
Acetylsalicylic acid (B01AC06)	75 (2.2)	27 (1.3)	21 (5.8)	3 (1.4)	24 (8.3)	0 (0.0)
Medications for constipation (A06)	90 (2.6)	22 (0.9)	23 (6.4)	13 (6.2)	24 (8.3)	8 (4.6)
Lactulose (A06AD11)	36 (1.1)	8 (0.3)	3 (0.8)	1 (0.5)	9 (3.1)	1 (0.6)
Medications for functional gastrointestinal disorders (A03)	85 (2.5)	15 (0.6)	32 (8.9)	12 (5.7)	18 (6.2)	8 (4.6)
Metoclopramide (A03FA01)	25 (0.7)	11 (0.5)	8 (2.2)	3 (1.4)	1 (0.3)	2 (1.1)
Medications for obstructive airway diseases (R03)	79 (2.3)	24 (1.0)	20 (5.6)	4 (1.9)	23 (7.9)	8 (4.6)
Salbutamol (R03AC02)	24 (0.7)	8 (0.3)	2 (0.6)	0 (0.0)	6 (2.1)	4 (2.3)
Thyroid therapy (H03)	74 (2.2)	19 (0.8)	28 (7.8)	12 (5.7)	12 (4.1)	3 (1.7)
Levothyroxine sodium (H03AA01)	73 (2.1)	18 (0.8)	28 (7.8)	12 (5.7)	12 (4.1)	3 (1.7)
Antibacterials for systemic use (J01)	59 (1.7)	25 (1.1)	14 (3.9)	5 (2.4)	10 (3.4)	5 (2.9)
Not specified	26 (0.8)	11 (0.5)	7 (1.9)	0 (0.0)	7 (2.4)	1 (0.6)
Sex hormones and modulators of the genital system (G03)	55 (1.6)	30 (1.3)	9 (2.5)	6 (2.9)	2 (0.7)	8 (4.6)
Not specified	29 (0.4)	25 (1.1)	0 (0.0)	4 (1.9)	0 (0.0)	0 (0.0)

Supplemental Table S3. The ten most reported ATC classes and individual medications within each class among post-partum women according to country.

Medication Class	Any Use (N=1799)	Norway (N=1113)	Belgium (N=235)	Switzerland (N=176)	United Kingdom (N=120)	Netherlands (N=155)
Analgesics (N02)	791 (44.0)	481 (43.2)	125 (53.2)	67 (38.1)	55 (45.8)	63 (40.6)
Paracetamol (N02BE01)	630 (35.0)	425 (38.2)	100 (42.6)	34 (19.3)	25 (20.8)	46 (29.7)
Antiinflammatory and antirheumatic products (M01)	300 (16.7)	194 (17.4)	52 (22.1)	24 (13.6)	22 (18.3)	8 (5.2)
Ibuprofen (M01AE01)	257 (14.3)	161 (14.5)	49 (20.9)	24 (13.6)	20 (16.7)	3 (1.9)
Antihistamines for systemic use (R06)	216 (12.0)	171 (15.4)	24 (10.2)	6 (3.4)	6 (5.0)	9 (5.8)
Cetirizine (R06AE07)	73 (4.1)	65 (5.8)	6 (2.6)	0 (0.0)	1 (0.8)	1 (0.6)
Drugs for acid related disorder (A02)	214 (11.9)	103 (9.3)	45 (19.2)	14 (8.0)	27 (22.5)	25 (16.1)
Not specified	59 (3.3)	4 (0.4)	18 (7.7)	10 (5.7)	15 (12.5)	12 (7.7)
Antibacterials for systemic use (J01)	168 (9.3)	120 (10.8)	10 (4.3)	10 (5.7)	17 (14.2)	11 (7.1)
Not specified	87 (4.8)	65 (5.8)	5 (2.1)	6 (3.4)	6 (5.0)	5 (3.2)
Medications for constipation (A06)	167 (9.3)	77 (6.9)	42 (17.9)	18 (10.2)	15 (12.5)	15 (9.7)
Lactulose (A06AD11)	52 (2.9)	46 (4.1)	3 (1.3)	0 (0.0)	2 (1.7)	1 (0.6)
Medications for obstructive airway diseases (R03)	70 (3.9)	57 (5.1)	3 (1.3)	3 (1.7)	5 (4.2)	2 (1.3)
Salbutamol (R03AC02)	18 (1.0)	17 (1.5)	0 (0.0)	0 (0.0)	0 (0.0)	1 (0.6)
Thyroid therapy (H03)	68 (3.8)	39 (3.5)	22 (9.4)	4 (2.3)	3 (2.5)	0 (0.0)
Levothyroxine sodium (H03AA01)	68 (3.8)	39 (3.5)	22 (9.4)	4 (2.3)	3 (2.5)	0 (0.0)
Antithrombotic agents (B01)	64 (3.6)	37 (3.3)	9 (3.8)	4 (2.3)	8 (6.7)	6 (3.9)
Enoxaparin (B01AB05)	18 (1.0)	11 (1.0)	4 (1.7)	2 (1.1)	0 (0.0)	1 (0.6)
Beta blocking agents (C07)	46 (2.6)	36 (3.2)	3 (1.3)	1 (0.6)	3 (2.5)	3 (1.9)
Labetalol (C07AG01)	38 (2.1)	29 (2.6)	3 (1.3)	0 (0.0)	3 (2.5)	3 (1.9)

Supplemental Table S4. The ten most reported ATC classes of chronic medications changed on women's own initiative due to the pandemic (N= 2223).

**Medication Classes (ATC level 2)**	**N (%)**
Analgesics (N02)	26 (1.2)
Antihistamines for systemic use (R06)	19 (0.9)
Anti-inflammatory and antirheumatic products (M01)	9 (0.4)
Medications for obstructive airway diseases (R03)	7 (0.3)
Nasal preparations (R01)	7 (0.3)
Psychoanaleptics (N06)	6 (0.3)
Corticosteroids, dermatological preparations (D07)	4 (0.2)
Antiepileptics (N03)	3 (0.1)
Immunosuppressants (L04)	2 (0.1)
Medications used in diabetes (A10)	2 (0.1)
Agents acting on the RAA system (C09)	2 (0.1)
Muscle relaxants (M03)	2 (0.1)
Corticosteroids for systemic use (H02)	2 (0.1)

Results are expressed as absolute numbers (%).

Supplemental Table S5. Reasons for changing the use of a chronic medication due to the COVID-19 pandemic.

Medication (ATC Level 5)	Reason Category (N)	Reasons in Relation to COVID-19	Country of Respondent
Amlodipine (C08CA01)	Limited access to a healthcare professional/service (3)	"Couldn't go to my GP for a phasing-out schedule, so I stopped on my own initiative because my blood pressure was structurally low."	Netherlands
Candesartan (C09CA06), Rizatriptan (N02CC04)		"Because the medications are not good when you are pregnant and it took time to get in touch with a doctor, I decided to stop them myself."	Norway
Nafarelin (H01CA02)		"I had no contact with the hospital because of the strike and the coronary pandemic. Had to stop using Synarela, which I was using for infertility treatment. Had to stop on my own since I had no contact with the hospital and I became spontaneously pregnant."	Norway
Mometasone (R01AD09)	Fear of contracting the disease / preventive measure (6).	"Increased use of allergy medications to avoid respiratory symptoms that others may misinterpret as symptoms of covid-19 infection."	Norway

Salmeterol and fluticasone (R03AK06)		"I increased the dose, in fear of not getting well and in fear of increasing the contagion while being sick"	Norway
Budesonide (R03BA02)		"Preventive measures to avoid respiratory infections in the brain"	Norway
Doxylamine, combinations (R06AA59)		"To avoid people at the doctor's"	Belgium
Cetirizine (R06AE07)		"Due to the pandemic, I prefer to keep my asthma and allergies under control"	Norway
Non specified		"Because I was worried about my immune system if I became infected"	
L04AB05 (Certolizumab)	Stress and uncertainty (2)	"Stopping the medication due to uncertainty at the start of the pandemic"	Norway
Botulinum toxin (M03AX01)		"Want to keep me as calm as possible during the pandemic."	Netherlands
Desloratadine (R06AX27)	Fear of public stigmatization (2).	"Took more allergy medication to avoid sneezing in public. And for hygienic reasons, less runny nose and itchy eyes make me touch my face less often"	Norway

Cetirizine (R06AE07)		"I increased the use of allergy medications to avoid "respiratory symptoms" that other may misinterpret as symptoms of covid-19"	Norway
Rituximab	Vaccine side-effects (2).	"Got vaccine, must wait to take next dose of rituximab"	Norway
Paracetamol (N02BE01)		" Taken only after vaccination, as soon as fever was gone also stopped the medication"	Belgium

Supplemental Table S6. Comparison between the general birthing population and the study sample of pregnant and breastfeeding women for each country.

	BELGIUM			NORWAY			THE NETHERLANDS			SWITZERLAND			UK		
	**General** [a]	**Preg (n=360)**	**PP (n=235)**	**General** [b]	**Preg (n=2376)**	**PP (n=1113)**	**General** [c]	**Preg (n=175)**	**PP (n=155)**	**General** [d]	**Preg (n=210)**	**PP (n=176)**	**General** [e]	**Preg (n=290)**	**PP (n=120)**
Maternal age (years)															
18-25	10.1	2.2	3.0	8.4	8.2	7.5	8.3	5.1	0.0	7.5	0.5	0.6	22.7	3.8	2.5
26-30	34.1	33.6	25.1	30.7	38.8	40.0	29.3	19.4	20.0	26.4	15.2	14.2	28.6	14.1	17.5
31-35	37.2	37.5	40.0	38.4	39.1	39.6	39.9	30.3	33.5	39.9	36.2	37.5	29.4	37.6	25.8
36-40	15.2	5.6	7.7	18.3	12.2	11.0	18.9	9.7	14.2	21.8	19.0	19.3	15.7	13.1	14.2
> 40	3.4	1.1	1.3	4.1	1.7	2.0	3.7	3.4	4.5	4.4	4.3	4.5	3.6	3.1	3.3
Relationship status															
Partner	81.5	79.2	74.9	94.2	97.9	98.7	91.0	66.3	71.6	83.2	75.2	75.6	94.8	71.4	63.3
No partner	19.5	0.8	2.1	5.8	2.1	1.3	9.0	1.7	0.6	16.8	0.0	0.6	5.2	0.3	0.0
Professional status															
Professionally active	75.3	76.1	71.5	76.5	87.4	87.8	82.0	62.9	67.1	82.5	69.0	67.6	72.8	66.6	51.7
Education level															
Low	12.0	0.6	0.0	24.0	2.9	2.2	10.7	1.1	3.9	42.7	0.5	0.6	45.7	0.70	2.5
Medium	40.0	11.7	12.3	36.2	17.0	17.3	33.8	16.6	10.3	12.0	17.6	19.9	18.6	10.0	15.0
High	48.0	67.5	64.3	39.8	80.3	80.6	53.6	49.7	58.1	45.3	48.6	49.4	30.4	61.0	45.0
Smoking in pregnancy/ post-partum**															
Yes	6.0	1.4	3.0	2.4/1.4[f]	0.8	1.2	7.4	2.3	1.3	7.0	4.3	3.4	12.4	1.7	2.5
No	94.0	78.6	74.0	97.6/98.6[f]	99.2	98.8	92.6	65.7	71.0	93.0	71.0	72.7	87.6	70.0	60.8

Preg = pregnant; PP = postpartum. Data are presented as %. N/A = not available. Numbers may not add up due to missing values. aBELGIUM: Statistics for the general birthing population were retrieved from Statbel (education level (25-44 years, 2019); professional status (25-49 years, 2016)), SPE (maternal age, 2020) and Sexpert (relationship status (18-39 years, 2013)). Age categories reported in the perinatal statistics report differ slightly from those reported in the table: 40.

Smoking in pregnancy/breastfeeding has been estimated in the publication with PMID 31342605. bNORWAY: Statistics for the general birthing population in Norway were retrieved from the Medical Birth Registry of Norway in 2020 (age, relationship status, parity and smoking) (http://statistikkbank.fhi.no/mfr/). Official statistics from the Norwegian Medical Birth Registry have a slightly different age categorization; 18 to 24 years, 25 to 29 years, 30 to 34 years, 35 to 39 years, 40 to 44 years. Numbers reported follow this categorization. Statistics on educational level (women aged 16 years and older) were retrieved from from Statistics Norway in 2020 (https://www.ssb.no/en/utdanning/utdanningsniva/statistikk/befolkningens-utdanningsniva); Low = below upper secondary education; Medium = upper secondary education & vocational education; High = higher education. Statistics on professional status (women aged 25 – 39 years) were retrieved from Eurostat statistics (https://ec.europa.eu/eurostat/). cTHE NETHERLANDS: Statistics for the general birthing population in the Netherlands were retrieved from Perined, the perinatal registry of the Netherlands (data for 2019) (maternal age, parity), from CBS StatLine, general statistics Netherlands (data for 2020) (education level, professional status and relationship status (2017)); and from Trimbose Institute for mental health (data for 2018) (smoking in pregnancy). dSWITZERLAND: Statistics for the general birthing population in Switzerland were retrieved from the Federal Statistical Office (FSO) in 2020 for maternal age, relationship status (aged 25-44 years), professional status (aged 25-54 years), education level (aged 25–34 years) and 2018 for parity (aged 25–44 years) (https://www.bfs.admin.ch). Smoking in pregnancy in Switzerland has been monitored on behalf of FSO between 2011 and 2016 (https://www.infoset.ch/fr/tabac.html). eUK: Statistics for the general birthing population are retrieved from various datasets provided by: (i) the Office of National Statistics including Conception Statistics for England and Wales (maternal age and relationship status – data from 2018), Labour Market Statistics (educational attainment in men and women aged 16-49 – data from 2011, and professional status in women aged 16-64 – data from Jan to Jun 2020) and Adult Smoking Habits in England (women aged 16-49 – data from 2019); (ii) NHS Digital Hospital Episode Statistics NHS Maternity Statistics (smoking at booking appointment – data from 2018- 19); and (iii) population-based published estimates (maternal parity – PMID: 30464639). f start of pregnancy / end of pregnancy. Missing data: Belgium (23.0%), Switzerland (23.9% - 24.4%), The Netherlands (27.7%), The UK (36.7% - 43.3%).
